# The time is ripe for robopsychology

**DOI:** 10.3389/fpsyg.2022.968382

**Published:** 2022-09-22

**Authors:** Christian U. Krägeloh, Jaishankar Bharatharaj, Jordi Albo-Canals, Daniel Hannon, Marcel Heerink

**Affiliations:** ^1^PAIR Lab New Zealand, Auckland University of Technology, Auckland, New Zealand; ^2^PAIR Lab India, Bharath Institute of Higher Education and Research, Chennai, Tamil Nadu, India; ^3^Lighthouse Disruptive Innovation Group, LLC., Cambridge, MA, United States; ^4^Department of Mechanical Engineering, Tufts University, Medford, MA, United States; ^5^Saxion University of Applied Sciences, Enschede, Netherlands

**Keywords:** psychology, sub-discipline, special interest group, robot psychology, robotic psychology, robopsychology, robots, artificial intelligence

## Abstract

As robotic applications become increasingly diverse, more domains of human lives are being involved, now also extending to educational, therapeutic, and social situations, with a trend to even more complex interactions. This diversity generates new research questions that need to be met with an adequate infrastructure of psychological methods and theory. In this review, we illustrate the current lack of a sub-discipline in psychology to systematically study the psychological corollaries of living in societies where the application of robotic and artificial intelligence (AI) technologies is becoming increasingly common. We thus propose that organized efforts be made toward recognition of robopsychology as a sub-discipline so that the field of psychology moves away from isolated publications of robot- and AI-related topics to a body of knowledge that is able to meet the demands for change, as the world is preparing for the Fourth Industrial Revolution. We propose a definition of robopsychology that not only covers the study of the effects of robots on human behavior, but also of robots and AI themselves, as well as acknowledging how this sub-discipline may eventually be fundamentally changed through robots and AI. In this sense, our definition mirrors an already existing definition of the field of robophilosophy.

## Introduction

While the word *robot* has only first appeared in the early 1920s through Karel Čapek’s science fiction play *R.U.R.* (Rossum’s Universal Robots; Čapek, 2004), the idea of self-moving machines, or automata, has featured in myths and stories that go back three millennia and are found in many parts of the world (Mayor, 2018). Throughout history, there have also been many independent attempts to create actual automata, such as water-powered organs or mechanized beasts and androids (Cave and Dihal, 2018). Of course, it was not until the rapid technological progress of the 20^th^ century that robots became more wide spread. For example, the adoption of robotic technology in the automotive manufacturing industry resulted in dramatic increases in cost-efficiency and production quality (Karabegović, 2016). Due to the precision they provide, robots have also become commonplace in medical contexts such as in surgery (Lane, 2018).

Most individuals would rarely encounter industrial and surgical robots, and if so, only witness the very specific functions that these robots provide. This is in contrast with the notion of robots as embodied intelligent and autonomous agents and particularly with portrayal in media and film, where robots often appear as highly sophisticated and with the potential to lead to utopian or dystopian scenarios (Mubin et al., 2019). Fact-based media reporting has been shown to increase positive attitudes and trust in robots (Savela et al., 2021), and actual encounters with robots also have the potential to alleviate much of the anxiety and wariness that people may have. While instances of robotic hotel check-in and room service (Fuentes-Moraleda et al., 2020) or robotic chefs in restaurants (Fusté-Forné, 2021) may still be viewed as having primarily entertainment value, systematic attempts have increasingly been made to apply robots to provide psychosocial or educational benefits for humans. Robots have thus been used to provide companionship for older people (Gasteiger et al., 2021), robot-enhanced psychotherapy (Costescu et al., 2014), or to assist in learning and teaching (Belpaeme et al., 2018). As human-robot interactions are appearing to become more lively and reciprocal, more effort is directed at studying the psychological reactions of human users in order to optimize this experience. Research has thus explored the effects of a range of variables such as robot morphology (Mara et al., 2022), voice (Dong et al., 2021), or nonverbal behavior (Zinina et al., 2020). Recent research has even explored the extent to which what the appearance of robots may be racialized with the potential to perpetuate racial stereotypes (Bartneck et al., 2018).

The trend towards increased relevance of robots in people’s lives accelerates the need to understand the variables that influence the quality of human-robot interactions as well as their psychological corollaries. While a solid body of research has already emerged (Siciliano and Khatib, 2016), new research questions continue to be posed, particularly the extent to which such applications are motivated by or fulfil humans’ psychological needs. As shown by robotic pets (Melson et al., 2009), robotic romance (Viik, 2020), sex robots (Döring et al., 2020), or robots to provide spiritual and religious support (Trovato et al., 2021), human-robot interactions are increasing in complexity, thus connecting robot research with the same psychological models and theories that are used to explain social behaviors among humans, such as attachment theory (Pozharliev et al., 2021) or social identity theory (Edwards et al., 2019). The purpose of the present review was to explore the extent to which there are any existing sub-disciplines in psychology devoted to the study of topics involving robots. Using a state-of-the-art review approach (Grant and Booth, 2009) with a systematic search strategy, we provided an outline of the landscape of psychological sub-disciplines. Not being based on any previous theories or hypotheses, this review followed an inductive approach (Watson et al., 2018).

## A review of the representation of the study of robots in existing sub-disciplines of psychology

At the time of writing this review (May 2022), the journal *Frontiers in Psychology* listed 32 sub-disciplines or sub-fields of psychology to structure the content of its articles (Frontiers in Psychology, 2022). We present these in Table 1, together with potential sub-discipline names expressed through the 54 divisions recognized by the American Psychological Association (APA) at the time of writing this review (APA, 2022a). APA notes that some of the divisions represent special interest groups rather than sub-disciplines. However, for the purposes of identifying representation of robotics-related research in psychology, including special interest groups in addition to sub-disciplines provides a more comprehensive analysis. Additionally, we searched through the APA literature database PsycInfo for journal titles that could indicate a sub-discipline that may have recently emerged or is too small to have been recognized yet as a sub-field in psychology. We searched this psychology database containing nearly 2,300 journals for the word stem “psycho” to identify potential sub-discipline names that are expressed either by a preceding adjective other than a geographical location (e.g., applied psychology), a preceding noun (e.g., community psychology), or by a prefix (e.g., ecopsychology). The presence of two adjectives was considered to be too specific and indicative of a further sub-categorization within a sub-discipline. For example, *applied social psychology* was not included as it was treated as a further division of *social psychology*. If a name contained two adjectives (e.g., *reproductive and infant psychology*), the entry was presented like that, unless both adjectives had already resulted in a separate entry. Synonyms or very similar terms were still retained as separate entries, such that both *child psychology* and *pediatric psychology* were included. The search was conducted by the first author using coding for relevance, which was verified independently by the second author. Any uncertainty was resolved by discussion. In total, we list 127 entries in Table 1, with information on where they were sourced from.

**Table 1:** Sub-disciplines and special interest groups of psychology as presented by *Frontiers in Psychology*, *APA*, and in academic journal titles.

Sub-discipline	Source
Addiction Psychology	APA Division 50
Advancement of Psychotherapy	APA Division 29
Aerospace Psychology	Journal name “The International Journal of Aerospace Psychology”
Aging Psychology	Journal name “Aging Psychology”
American Psychology-Law Society	APA Division 41
Analytical Psychology	Journal name “The Journal of Analytical Psychology”
Animal Psychology	Journal name “Japanese Journal of Animal Psychology”
Auditory Cognitive Neuroscience	Frontiers in Psychology section
Adult Development and Aging	APA Division 20
Applied Experimental and Engineering Psychology	APA Division 21
Applied Psychology	Journal name “Journal of Applied Psychology”
Aviation Psychology	Journal name “Aviation Psychology and Applied Human Factors”
Behavioral Neuroscience and Comparative Psychology	APA Division 6
Behavioral Psychology	Journal name “Behavioral Psychology”
Behavior Analysis	APA Division 25
Biological Psychology	Journal name “Biological Psychology”
Black Psychology	Journal name “Journal of Black Psychology”
Child and Family Policy and Practice	APA Division 37
Child and Adolescent Psychology	Journal name “Journal of Clinical Child and Adolescent Psychology”
Child Psychology	Journal name “Educational and Child Psychology”
Clinical Child and Adolescent Psychology	APA Division 53
Clinical Neuropsychology	APA Division 40
Clinical Psychology	APA Division 12
Coaching Psychology	Journal name “International Coaching Psychology Review”
Cognition	Frontiers in Psychology section
Cognitive Psychology	Journal name “Cognitive Psychology”
Cognitive Science	Frontiers in Psychology section
Community Psychology	APA Division 27
Comparative Psychology	Frontiers in Psychology section
Consciousness Research	Frontiers in Psychology section
Constructivist Psychology	Journal name “Journal of Constructivist Psychology”
Counseling Psychology	APA Division 17
Consulting Psychology	APA Division 13
Consumer Psychology	APA Division 23
Couple and Family Psychology	APA Division 43
Cross-Cultural Psychology	Journal name “Journal of Cross-Cultural Psychology”
Cultural Psychology	Frontiers in Psychology section
Cyberpsychology	Journal name “Cyberpsychology, Behavior, and Social Networking”
Decision Neuroscience	Frontiers in Psychology section
Developmental Psychology	APA Division 7; Frontiers in Psychology section
Eating Behavior	Frontiers in Psychology section
Ecological Psychology	Journal name “Ecological Psychology”
Economic Psychology	Journal name “Journal of Economic Psychology”
Ecopsychology	Journal name “Ecopsychology”
Educational Psychology	APA Division 15; Frontiers in Psychology section
Emotion Science	Frontiers in Psychology section
Environmental, Population and Conservation Psychology	APA Division 34
Environmental Psychology	Frontiers in Psychology section
Ethnic Minority Psychology	Journal name “Cultural Diversity and Ethnic Minority Psychology”
Evolutionary Psychology	Frontiers in Psychology section
Experimental Psychology and Cognitive Science	APA Division 3
Family Psychology	Journal name “Journal of Family Psychology”
Forensic and Legal Psychology	Frontiers in Psychology section
Forensic Psychology	Journal name “American Journal of Forensic Psychology”
Gender, Sex and Sexualities	Frontiers in Psychology section
General Psychology	APA Division 1
Genetic Psychology	Journal name “The Journal of Genetic Psychology: Research and Theory on Human Development”
Gerontopsychology	Journal name “GeroPsych: The Journal of Gerontopsychology and Geriatric Psychiatry”
Group Psychology and Group Psychotherapy	APA Division 49
Health Psychology	APA Division 38; Frontiers in Psychology section
Health Service Psychology	Journal name “Journal of Health Service Psychology: An Official Journal of the National Register of Health Service Psychologists”
History of Psychology	APA Division 26
Humanistic Psychology	APA Division 32
Human-Media Interaction	Frontiers in Psychology section
Individual Psychology	Journal name “The Journal of Individual Psychology”
Industrial and Organizational Psychology	APA Division 14
Intellectual and Developmental Disabilities/Autism Spectrum Disorder	APA Division 33
International Psychology	APA Division 52
Investigative Psychology	Journal name “Journal of Investigative Psychology and Offender Profiling”
Language Sciences	Frontiers in Psychology section
Latinx Psychology	Journal name “Journal of Latinx Psychology”
Legal and Criminological Psychology	Journal name “Legal and Criminological Psychology”
Managerial Psychology	Journal name “Journal of Managerial Psychology”
Mathematical Psychology	Journal name “Journal of Mathematical Psychology”
Mathematical and Statistical Psychology	Journal name “British Journal of Mathematical and Statistical Psychology”
Media Psychology and Technology	APA Division 46
Medical Psychology	Journal name “Medizinische Psychologie” [German]
Military Psychology	APA Division 19
Movement Science and Sport Psychology	Frontiers in Psychology section
Neuropsychology	Frontiers in Psychology section
Occupational and Organizational Psychology	Journal name “Journal of Occupational and Organizational Psychology”
Organizational Psychology	Frontiers in Psychology section
Pastoral Psychology	Journal name “Pastoral Psychology”
Peace Psychology	APA Division 48
Pediatric Psychology	APA Division 54; Frontiers in Psychology section
Perception Science	Frontiers in Psychology section
Performance Science	Frontiers in Psychology section
Personality and Social Psychology	APA Division 8; Frontiers in Psychology section
Personnel Psychology	Journal name “Personnel Psychology”
Phenomenological Psychology	Journal name “Journal of Phenomenological Psychology”
Philosophical Psychology	Journal name “Philosophical Psychology”
Police and Criminal Psychology	Journal name “Journal of Police and Criminal Psychology”
Political Psychology	Journal name “Political Psychology”
Positive Psychology	Frontiers in Psychology section
Prescribing Psychology	APA Division 55
Professional Psychology	Journal name “Professional Psychology: Research and Practice”
Projective Psychology	Journal name “Journal of Projective Psychology & Mental Health”
Psychoanalysis and Psychoanalytic Psychology	APA Division 39
Psychological Hypnosis	APA Division 30
Psychological Study of Culture, Ethnicity and Race	APA Division 45
Psychological Study of Men and Masculinities	APA Division 51
Psychological Study of Social Issues	APA Division 9
Psychologists in Independent Practice	APA Division 42
Psychologists in Public Service	APA Division 18
Psychology for Clinical Settings	Frontiers in Psychology section
Psychology of Aging	Frontiers in Psychology section
Psychology of Aesthetics, Creativity and the Arts	APA Division 10
Psychology of Religion and Spirituality	APA Division 36
Psychology of Sexual Orientation and Gender Diversity	APA Division 44
Psychology of Women	APA Division 35
Psycho-Oncology	Frontiers in Psychology section
Psychopharmacology and Substance Abuse	APA Division 28
Psychopathology	Frontiers in Psychology section
Qualitative Psychology	Journal name “Qualitative Psychology”
Quantitative and Qualitative Methods	APA Division 5
Quantitative Psychology and Measurement	Frontiers in Psychology section
Reading Psychology	Journal name “Reading Psychology”
Rehabilitation Psychology	APA Division 22
Reproductive and Infant Psychology	Journal name “Journal of Reproductive and Infant Psychology”
School Psychology	APA Division 16
Social Psychology	Journal name “Social Psychology”
Sport, Exercise and Performance Psychology	APA Division 47
State, Provincial and Territorial Psychological Association Affairs	APA Division 31
Teaching of Psychology	APA Division 2
Theoretical and Philosophical Psychology	APA Division 24; Frontiers in Psychology section
Transpersonal Psychology	Journal name “Journal of Transpersonal Psychology”
Trauma Psychology	APA Division 56

The entries are listed in alphabetical order. For journal titles, representative examples are shown.

None of the 127 entries in Table 1 make any reference to robots. APA Division 21 (*Applied Experimental and Engineering Psychology*) might initially appear to have some relevance to robotics but is very broadly worded as promoting “the development and application of psychological principles, knowledge, and research to improve technology, consumer products, energy systems, communication and information, transportation, decision making, work settings and living environments” (APA, 2022b). While three journal titles in PsycInfo contained the word “robot,” none of these are representative of what may be considered a relevant sub-discipline of psychology. *ACM Transaction of Human-Robot Interaction* is described on its homepage (Association for Computing Machinery, 2022) to be an interdisciplinary journal that also welcomes submissions from behavioral and social sciences. *Intelligent Service Robotics* (Springer, 2022a) is focused on assistive functions of robots, making some mention of the relevance of cognitive science, and *International Journal of Social Robotics* (Springer, 2022b) is presented as an interdisciplinary journal that does not mention psychology specifically.

## Discussion: Robot psychology, robotic psychology, or robopsychology?

The list in Table 1 indicates that there is currently no sub-discipline in psychology that can be considered to be giving robots special attention, either as experimental subjects or by studying their effects on human behavior. Of course, this does not mean that a potential psychological sub-discipline may not already have some sort of presence in the literature through individual publications. What are some potential sub-discipline names mentioned in this work and what do these names suggest about the way in which robots are studied? When searching the academic literature (using GoogleScholar) for “robot psychology,” a small number of articles can be found. This includes a technical note by Konolige (1985) where *experimental robot psychology* is purported to be about “analyzing the design of a robot agent’s cognitive processes” (p. 2). Gallagher (2013) referred to robot psychology when describing a robot’s understanding of its own propositional attitudes (as equivalent to folk psychology for humans), and Nitsch and Popp (2014) used the term in the context of describing how robots as social agents need to be able to “predict human intentions and actions and display behavior that is appropriate to that context” (p. 622). Therefore, just like animal psychology is about understanding the behavior of animals, robot psychology is focused on robots only and thus not aspects related to the human perspective when interacting with robots.

A suitable alternative to *robot psychology* is *robotic psychology*. While this phrase has also been mentioned only very little in the literature, it has been clearly defined as the study of “individual differences in people’s interactions with various robots, as well as the diversity of the robots themselves, applying principles of differential psychology to the traditional fields of human factors and human–computer interactions” (Libin and Libin, 2004, p. 1792). The authors contrasted robotic psychology with *robopsychology*, which they defined as “a systematic study of compatibility between people and artificial creatures” as well as the study of “psychological mechanisms of the animation of the technological entity which result in a unique phenomenon defined as a robot’s ‘personality’” (p. 1792). Unlike robotic psychology, which “focuses on the psychological significance of person-robotic creature communication” (Libin and Libin, 2004, p. 1792), the focus of robopsychology is thus on the understanding of robot behavior. This usage of the term is consistent with how it was first used when introduced as the name of a fictional science in short stories by Isaac Asimov in 1950 (Bátfai, 2020).

While some studies (Servick, 2019) have interpreted the term robopsychology in a way consistent with the definition above, other researchers have used the term interchangeably with robotic psychology (Duradoni et al., 2021; Linz Institute of Technology, 2022). In the absence of any well-established or consistent use of any of these terms, a future sub-discipline in psychology related to robots may still decide on a suitable name. In our view, the term *robopsychology* is preferable as it can be easily identified alongside the already established field of *robophilosophy* (Tzafestas, 2016) – the “philosophy *of*, *for*, and *by* social robotics” (Seibt, 2018, p.390). Philosophy *of* social robotics is seen as the reflective activities about conceptual implications of investigating human-robot interactions, while philosophy *for* reflects on conceptual norms, sociality, human capacities, social roles as well as legal and ethical responsibilities, and philosophy *by* expresses any fundamental re-orientation of philosophical research that might occur due to its activities (Seibt, 2018).

The tentative definition of robopsychology that we would like to offer is similar: the psychology *of*, *for*, and *by* robots, robotics, and artificial intelligence (AI). This wording contains a broader scope than social robots only. Additionally, *robots and robotics* expresses the fact that both the actual products as well as the ongoing process of designing and building robots are worthy topics for psychological research. We also propose to add AI so that the sub-discipline is not only limited to physical manifestations but also considers latent processes related to this technology. In this definition, the psychology *of* robots, robotics, and AI addresses psychological implications of encountering robots and AI as well as people’s views regarding this technology. Psychology *for* concerns areas that are relevant in the design of robots and AI and the facilitation of the robotic applications in society. Lastly, psychology *by* acknowledges any fundamental changes in the way in which psychological topics in the study of robots and AI may be approached in the future. The latter can include issues such as transhumanism (DeFalco, 2020) and expresses the potential for AI to eventually even participate in the discipline of psychology.

## Conclusion: The need for a science of robopsychology

With the predicted arrival of the so-called Fourth Industrial Revolution characterized by transformation through robotics and automation (Karabegović et al., 2020), psychological research can be expected to experience transformational changes. A rapidly expanding scope of application of robotic technology is already noticeable as robotics has moved from primarily industrial uses to areas involving direct contact with people, such as robots in the service industry, in educational settings, and as social agents. As our review illustrated, there is currently no psychological sub-discipline dedicated to the study of the effects that robots have on people’s lives, which is currently only addressed through interdisciplinary fields such as human-robot interaction or social robotics. The advantages of organizing psychological research through the formation of special interest groups and sub-disciplines is undoubtedly the driver of the richness and diversity demonstrated in Table 1 of our review. With this review, we encourage activities toward the recognition of robopsychology as the sub-discipline that enables the necessary academic and theoretical infrastructure to facilitate psychological investigations in this changing world. Such work requires specific psychological theories and models to describe the increasing complexities of human interactions with robots, such as intimacy and spirituality, as well as suitable research methods and measurement of psychological constructs that meet quality standards for psychological research (Krägeloh et al., 2019). Our proposed definition of robopsychology is intentionally broad to permit a range of future applications and may be considered parallel to the already existing sub-discipline of robophilosophy. To what extent there is eventual demand for the sub-discipline of robopsychology is up for the future to decide. With this article, we hope to instigate the necessary debates.

## Author contributions

JB conceived of the idea of proposing the field of robopsychology, which was subsequently discussed by all authors. CK created the proposed definition for the field of robopsychology, conducted the review, and provided the first draft. All authors contributed to the article and approved the submitted version.

## Conflict of interest

JA-C was employed by the company Lighthouse Disruptive Innovation Group, LLC.

The remaining authors declare that the research was conducted in the absence of any commercial or financial relationships that could be construed as a potential conflict of interest.

## Publisher’s note

All claims expressed in this article are solely those of the authors and do not necessarily represent those of their affiliated organizations, or those of the publisher, the editors and the reviewers. Any product that may be evaluated in this article, or claim that may be made by its manufacturer, is not guaranteed or endorsed by the publisher.
